# Surgery

**Published:** 1876-03

**Authors:** 


### II. Surgery.

1. *Parenchymatous Injections.* Dr. Wilde. (*Deutsche Zeitschrift f. Chir.*, vi. 3.)

The doctor gives the experience he had with injections of iodine and nitrate of silver. In regard to the solutions of iodine or tincture of iodine injected into the parenchyma of goitres, he can only confirm the happy results obtained by Lücke and others. Even the largest goitres disappeared in a comparatively short time; the patients did not exhibit any signs of iodine intoxication or of a dangerous reaction which sometimes has been noticed, from the rapid absorption of strumous matter.

The injections with solutions of nitrate of silver were found especially effective in tumors of a soft, cellular structure. In order to have a good success it is necessary that the lymphatic glands in the vicinity of the neoplasm and all internal organs should be still intact, that the tumor itself be well defined and penetrable by the injections all round its circumference, and that the growth should be thoroughly impregnated with the solutions. For it is very erroneous to expect any satisfaction from two or three injections, made into a tumor of the size of a fist. The solution used for the purpose, consists of one grain of nitrate of silver to one ounce of distilled water. To substantiate his remarks, Dr. W. reports the history of three cases, of which we select the third: S., aged over forty years, laborer, called on Dr. W. in the fall of 1872. He had an epithelial cancer of the left cheek, which extended from the intra-orbital foramen to the border of the lower jaw, and from the angle of the mouth to the anterior border of the ear. The neighboring lymphatic glands were not affected. The carcinomatous degeneration occupied the skin, the subcutaneous tissue and the superficial strata of the muscles, but the mucous lining of the cheek was not affected. The surface of the neoplasm was covered by wart-like excrescences and its margin was rigid and elevated like a mound. The patient positively declining to submit to an operation, the doctor tried the effect of the parenchymatous injections with a solution of nitrate of silver. The carcinoma was accessible from every side and a thorough impregnation of it was practicable. Daily injections were made for ten days in succession; then the patient went home, to return from time to time as directed by the doctor. At his third visit, he was pronounced cured; the place of the carcinoma was occupied by a firm, smooth, cicatricial tissue. The patient has been examined several times since; no recidive has been yet discovered.

2. *Intra-Cranial Aneurism.* Wm. E. Humble, M.D. (*London Lancet*, Dec., 1875.)

The chief object of the author of this paper is to draw attention "to the importance of practicing auscultation of the head, more frequently than we are in the habit of doing." He cites the two recorded cases in which intra-cranial aneurism was diagnosticated during life—one by Mr. Coe, and another by Mr. Jonathan Hutchinson. These cases, with the one related by Mr. Humble, lead one to the opinion that, at some time during the progress of the aneurism, the diagnosis of this disease may be made by auscultation.

3. *Case of Aortic Aneurism Successfully Treated by the Distal Ligature.* Thos. Annandale, F.R.S.E. (*Canada Med. and Surg. Journal*, Jan., 1876; *British Med. Journal.*)

The patient was 62 years of age, and had suffered from symptoms of thoracic aneurism for about six months—symptoms that were steadily increasing in severity. On admission to the infirmary there was observed an aneurismal tumor, pulsating strongly and passing up from behind the clavicle and sterno-clavicular articulation into the neck, as far as the cricoid cartilage. The trachea was displaced towards the left side, and the inner half of the clavicle and its articulation with the sternum were pushed forward by the underlying tumor. The bruit, dullness on percussion, a constant irritating cough, pains shooting up to the head, want of sleep, and progressive emaciation, were the symptoms present. After one month of rest in bed and the use of iodide of potassium, sixty grains, daily, he left the hospital with his symptoms somewhat relieved. The improvement was, however, of short duration. The symptoms having grown more troublesome, the patient was re-admitted.

The cervical portions especially showed an increase in size. It had spread higher up, and laterally, so as to overlap the site of the subclavian artery. After a careful consideration of the circumstances of the case, and observing that pressure on the right carotid artery, immediately above the cervical portion of the tumor, very much diminished the aneurismal pulsation, it seemed a favorable opportunity for practicing distal ligation. The position of the aneurism did not permit the subclavian artery to be ligatured; but there was just sufficient room to secure the common carotid above the cervical portion of the tumor. The ligation of the artery immediately under the ovoid-hyoid muscle was performed March 2nd, under the carbolic spray. The prepared catgut ligature was employed.

The immediate effect of the ligation was to almost stop the aneurismal pulsation, and to convert the strong pulsation into a kind of quivering motion. No local or constitutional disturbance followed the operation. The wound had healed on the 17th. The day after the operation the patient felt greatly relieved. He no longer himself felt the pulsation in the tumor, and the pains in his head and neck had disappeared. The pulsation in the aneurism was felt to be very feeble and the tumor itself was decidedly smaller.

Dec. 27th, his condition was as follows: General health good; no pain or other uneasiness; he could stoop freely without giddiness, and could go up and down stairs easily; the cervical portion was half an inch lower than before the operation; the tumor was much flatter and firmer; a very feeble pulsation could be felt; a bruit, less loud than formerly, could still be heard on all sides of the tumor. Mr. Annandale remarks that, although it can not be said that the aneurism was completely cured, there can be no doubt as to the great relief afforded by the operation. He sees in this case additional evidence in favor of distal ligature on suitable examples of otherwise incurable aneurisms.

The author of the paper has great faith in the use of iodide of potassium; and expresses the opinion that should a fair trial of this drug fail to give complete relief to the symptoms, the distal ligature of the carotid should be employed, provided the case be a suitable one for the operation. The test of the suitability of the case for operation being the effect which temporary pressure on the distal portion of the carotid has on the aneurismal pulsation, provided, of course, there be sufficient space to ligature the common carotid. Mr. Annandale and Mr. Holmes agree that operative interference should, in the first instance, be confined to the ligation of the carotid, ligation of the third part of the subclavian being resorted to if advisable, at a future stage of the progress of the case.

4. *Treatment of Fractures.* Dr. SCHWAB. (*The Canada Medical Record*, December, 1875.)

Dr. Schwab (Wurzburg) has found albumen, as in the white of egg, to answer as well as plaster of Paris. In addition to the whites of six to eight eggs, there will be needed an old linen sheet, from which a bandage of Scultetus can be cut, a piece of pasteboard, which is always at hand in the cover of an old book, and a roller bandage, from three to four yards in length. The bandage of Scultetus and pasteboard are first saturated with the albumen, and the bandage carefully applied, allowing the edges to slightly overlap. This bandage should reach to the joints above and below the fracture. The pasteboard is then smoothly adjusted to the part, and secured with the roller. The limb is kept in proper position by means of small bandages, or cushions of straw. No *shortening*, or other deformity, *ever* followed. The author of this paper claims to have used the method referred to; for twelve years with *complete success.* He further believes, that with this bandage the fracture unites, and the mobility of the joint returns, much earlier than with *any* other dressing. This very gratifying result is *ascribed to the curative action of albumen.*

(We do not doubt that the method above mentioned *can* be made efficient. That it is convenient, none will deny. There are, however, no clinical facts to support the assertion, that fractures of the extremities can be managed by this means alone with "complete success," or that by it "no shortening ever followed;" or that any "curative action" can be ascribed to albumen, as contained in the bandage of Scultetus and pasteboard.)

5. *Hydrocele Cured by Injections of Carbolic Acid.* Dr. RAHN. (*Allg. Med. Zeitung*, 1875, No. 102.)

Prof. Hueter (Greifswald) has, of late, been using two per cent. solutions of carbolic acid, instead of the tincture of iodine, as an injection for hydrocele. After the liquid was withdrawn, he injected seven grammes of the above solution, and left it in the sac of the tunica vaginalis. No pain and very slight swelling followed such injections, and the patient was not obliged to lie in bed, while a radical cure was accomplished just as certainly as by the use of tincture of iodine.

Dr. R. could confirm these facts by the happy success he had in a case of hydrocele, which, in November, he treated with an injection of carbolic acid. Neither during nor after the operation, was there the slightest pain; indeed, the patient felt so well that he could not be induced by any means to remain in bed, and the next morning he walked in his garden. On the second day, with a well-fitting suspensory bandage, he went about his business as usual. On the fifth day, there was no swelling and no tenderness; in fact, no sign of a hydrocele; and this has since continued.

6. *The Hydrostatics of the Catheter.* DR. ROBERT SOMMERVILLE. (*Edinburgh Med. Jour.; Braithwaite's Retrospect*, Jan., 1876.)

The mechanical disadvantage to the bladder, of having to raise the urine four or five inches above its own level, is easily illustrated when the female bladder is being emptied with a flexible catheter. The urine runs freely enough if the nozzle of the catheter be kept down, but the stream is at once arrested if the end of the instrument be raised a few inches.

By using a very long, flexible catheter, and bending down the outer half of it so as to make the urine leave the instrument at as low a level as it enters it within the bladder, we balance the column which is obstructing the evacuation with one which facilitates it, and the only thing required of the bladder, in this case, is to overcome the friction of the urine against the interior of the tube. By bending down the end of the catheter still further, we convert it into a syphon, the long leg of which is external, and, the descending column of urine more than balancing the ascending one, the urine, having once begun to flow, is bound to go on running until the bladder is entirely empty. The urine no longer requires to be pressed out, it is drawn out.

All that is required to convert the ordinary metallic catheter into a syphon, is to have its external extremity end with a downward curve, and to slip on to it the end of an India rubber tube. The tube must be considerably longer than the catheter, in order that it may form the long leg, and the catheter the short leg, of the syphon. When, therefore, the catheter, with the tube thus attached to it, is introduced into the bladder distended with urine, and the external end is bent down towards the patient's bed, the first gush of urine fills both catheter and India-rubber tube, and flows out at the extremity of the latter. We may now allow the catheter to assume the position that the elasticity of the patient's tissues, uninterfered with, assigns to it. The external end of the catheter will spring up, yet the urine will continue to flow; for the syphon having once commenced to act, the emptying of the bladder will go on until the whole of what it contains is drawn off.

This proposed method of using the catheter as a syphon is very simple, very convenient, and very clean; it completely empties the bladder; it reduces the irritation of the mucous membrane by the catheter to the least possible degree, and it avoids entirely the temptation one often has, when the urine has ceased to flow, to raise the patient into an erect or semi-erect position—a proceeding never devoid of danger with a metallic catheter in the bladder.

7. *Recto-Urethral Fistula.* ED. J. BERMINGHAM. (*N. Y. Med. Jour.*, Feb., 1876.)

A young man, suffering from a "clap," noticed a dull pain in the perinæum, and in a couple of days the parts became so tender that he was obliged to take to his bed, and finally he could not pass water. An attempt to pass the catheter caused so much pain that the patient fainted; still the physician succeeded in drawing off the urine, but the next morning the patient noticed a profuse yellow discharge from the urethra, and had a stinging pain in the perinæum, greatly aggravated upon urinating. A few days later, he noticed, for the first time, that some fæces came through the urethra when he made water. An examination revealed no stricture, but a great tenderness in the perinæum, and in the rectum over the somewhat enlarged prostate; the sound caused great pain when passing the prostate; spasmodic contraction of the sphincter ani; no solution of continuity of rectum discoverable by finger, but an examination made by a speculum revealed a fistulous opening about an inch from the sphincter, into which a probe could be passed until it touched a sound that had been introduced into the urethra. The whole sinus was thrice cauterized by means of a silver probe coated with fused nitrate of silver; the sphincter ani was ruptured; opiate suppositories were given, and perfect rest ordered in the recumbent position. Under this treatment the patient completely recovered in three weeks.

